# Caspase-14 Expression Impairs Retinal Pigment Epithelium Barrier Function: Potential Role in Diabetic Macular Edema

**DOI:** 10.1155/2014/417986

**Published:** 2014-07-09

**Authors:** Selina Beasley, Mohamed El-Sherbiny, Sylvia Megyerdi, Sally El-Shafey, Karishma Choksi, Ismail Kaddour-Djebbar, Nader Sheibani, Stephen Hsu, Mohamed Al-Shabrawey

**Affiliations:** ^1^Cellular Biology and Anatomy, Medical College of Georgia, Georgia Regents University (GRU), Augusta, GA 30912, USA; ^2^Oral Biology/Anatomy, College of Dental Medicine, GRU, Augusta, GA 30912, USA; ^3^Culver Vision Discovery Institute and Department of Ophthalmology, Medical College of Georgia, GRU, Augusta, GA 30912, USA; ^4^Department of Anatomy, Mansoura Faculty of Medicine, Mansoura University, Mansoura, Egypt; ^5^Department of Physiology, Medical College of Georgia, GRU and Charlie Norwood VA Medical Center, Augusta, GA 30912, USA; ^6^Departments of Ophthalmology and Visual Sciences and Biomedical Engineering, University of Wisconsin School of Medicine and Public Health, Madison, WI 53705, USA

## Abstract

We recently showed that caspase-14 is a novel molecule in retina with potential role in accelerated vascular cell death during diabetic retinopathy (DR). Here, we evaluated whether caspase-14 is implicated in retinal pigment epithelial cells (RPE) dysfunction under hyperglycemia. The impact of high glucose (HG, 30 mM D-glucose) on caspase-14 expression in human RPE (ARPE-19) cells was tested, which showed significant increase in caspase-14 expression compared with normal glucose (5 mM D-glucose + 25 mM L-glucose). We also evaluated the impact of modulating caspase-14 expression on RPE cells barrier function, phagocytosis, and activation of other caspases using ARPE-19 cells transfected with caspase-14 plasmid or caspase-14 siRNA. We used FITC-dextran flux assay and electric cell substrate impedance sensing (ECIS) to test the changes in RPE cell barrier function. Similar to HG, caspase-14 expression in ARPE-19 cells increased FITC-dextran leakage through the confluent monolayer and decreased the transcellular electrical resistance (TER). These effects of HG were prevented by caspase-14 knockdown. Furthermore, caspase-14 knockdown prevented the HG-induced activation of caspase-1 and caspase-9, the only activated caspases by HG. Phagocytic activity was unaffected by caspase-14 expression. Our results suggest that caspase-14 contributes to RPE cell barrier disruption under hyperglycemic conditions and thus plays a role in the development of diabetic macular edema.

## 1. Introduction

Diabetic retinopathy (DR) is the most common complication of diabetes and remains a major cause of preventable blindness worldwide [1, 2]. Anatomical and functional changes occur in the retina and retinal pigment epithelium prior to clinical symptoms of the disease and RPE plays a key role in the pathogenesis of DR [3–5]. Most of the research on the physiopathology of DR has been focused on the impairment of the neuroretina and the breakdown of the inner blood retinal barrier (BRB). By contrast, the effects of diabetes on the RPE have received less attention and also the molecular mechanisms responsible for these early changes in the RPE remain unclear [6].

The retinal pigment epithelium is densely pigmented hexagonal monolayer of cells located between the neural retina and choroid blood vessels [7]. RPE cells are joined together by junction adhesion (JA) molecules and tight junction (TJ) proteins such as occludin, claudins, and zonula occludens [8, 9] which is linked to the actin cytoskeleton. Integrity of TJ and JA is important to keep the subretinal space dry and preserve the outer retinal barrier [10]. RPE is also responsible for light absorption and phagocytosis of shed photoreceptor of outer segments [11, 12]. Furthermore, RPE secretes several factors, which are involved in maintaining normal retinal vascular homeostasis such as platelet-derived growth factor (PDGF) [13], pigment epithelium-derived factor (PEDF) [14], and vascular endothelial growth factor (VEGF) [15–17]. RPE cell malfunction is involved in many eye diseases including age related macular degeneration (AMD) [18, 19] and DR through production of inflammatory cytokines and caspase-mediated inflammatory and apoptotic pathways [20]. High glucose treatment of RPE cells leads to disruption in the levels of gap junction protein connexin and the TJ protein claudin-1, causing epithelial barrier dysfunction [21, 22].

Caspases exist as inactive proenzymes, activation of which requires proteolytic processing at conserved internal aspartic residues to generate a heterotetrameric enzyme consisting of two large and two small subunits [23, 24]. Caspase-14 is expressed and activated mainly in the epidermis [25] and in several tissues related to barrier function such as choroid plexus, hair follicles, epidermis, RPE, thymic Hassall's bodies, and keratinized oral epithelium [26, 27]. Caspase-14 is thought to be associated with epidermal barrier formation that protects against dehydration and ultraviolet radiation-induced apoptosis [25]. Recently we demonstrated that caspase-14 is expressed in the retina and different retinal cells under normal conditions, and increased expression of caspase-14 occurs in the retinas of diabetic human subjects and experimental diabetic mice, as well as in retinal microvascular cells cultured under high glucose conditions [27]. We also showed that caspase-14 overexpression induced apoptosis of retinal endothelial cells and pericytes suggesting that caspase-14 plays a role in the pathogenesis of DR via accelerating retinal microvascular cell death [27] which contributes to the breakdown of the inner retinal barrier [28].

In this study, we investigated the functional involvement of caspase-14 in RPE cells. Our data showed that caspase-14 is involved in barrier function of RPE cells but not in phagocytic function. We evaluated the effect of high glucose (HG) on caspase-14 expression in RPE cells and we examined the effect of caspase-14 expression or knockdown on the RPE cell barrier function, activation of other caspases, and apoptosis under normal and hyperglycemic conditions. This study revealed upregulation of caspase-14 in RPE by HG treatment and disruption of RPE barrier by caspase-14 expression and HG treatment and this was associated with a significant increase in the activity of caspase-1 and caspase-9. Furthermore, the effects of HG treatment on RPE cells barrier function and caspase-1 and caspase-9 activities were prevented by caspase-14 knockdown.

## 2. Material and Methods

### 2.1. Cell Culture

The ARPE-19 cells are available from the American Type Culture Collection (ATCC) (Manassas, Virginia, USA). They are cultured under standard conditions (37°C in a humidified chamber of 5% CO_2_ in Dulbecco's modified Eagle's medium: nutrient mixture F-12 (DMEM/F-12) (Thermo Scientific, Wyman, Massachusetts) supplemented with 10% fetal bovine serum, 10,000 U/mL penicillin, 10 mg/mL streptomycin sulfate, and 25 *μ*g/mL amphotericin (Cellgro, Manassas, Virginia) which was changed every 3 days. Cells were passaged weekly at a ratio of 1 : 10.

### 2.2. High Glucose Treatment of RPE Cells

RPE cells were grown until 70–80% confluent, and then the serum-free DMEM/F-12 was added to the cells for 24 h before switching to high or normal glucose treatment (30 mM or 5 mM D-glucose + 25 L-glucose, resp.). The cells were then incubated in 37°C in a humidified chamber of 5% CO_2_ for 5 days. After the incubation, the medium was removed and the cell lysates were used for Western blot analysis of caspase-14.

### 2.3. Caspase-14 Transfection

The preparation of caspase-14 expression vector and empty vector was previously described [27], and the cDNA sequences of the vectors were confirmed by DNA sequencing. ARPE-19 cells were transfected with the pCMV plasmid containing human caspase-14 cDNA, the empty vector, scrambled siRNA (Integrated DNA Technologies, Coralville, Iowa), and caspase-14 siRNA (Santa Cruz, Dallas, Texas), by electroporation using ECM 830 electroporation system (Harvard, Holliston, Massachusetts). ARPE-19 cells were grown in 75 cm^2^ flasks. When cells reached 90%–95% confluence, the cells were passaged and resuspended in 4 mL PBS (Life Technologies, Grand Island, New York) and placed in an electroporation cuvette and 1 *μ*g of DNA or RNA was added to the cuvette. The cuvette was placed in the ECM 830 and the electroporation was done at 450 V for 75 *μ*s, repeated twice with 100 ms intervals. The cell mixture was mixed and the electroporation was repeated. The cells were then reseeded in 75 cm^2^ flasks.

### 2.4. Assessment of Retinal Pigment Epithelial Cell Barrier Function

Integrity of RPE barrier is essential for normal retinal function and it is disrupted by hyperglycemia contributing to the pathogenesis of diabetic macular edema. Therefore, we assessed whether caspase-14 is implicated in RPE barrier function by studying the effect of modulating its expression on RPE permeability, transcellular electrical resistance, and cytoskeleton.

### 2.5. FITC-Dextran Flux Assay

ARPE-19 cells transfected with caspase-14 or empty vector were seeded on noncoated membranes with 0.4 *μ*m pores (Transwell; Corning Costar), in Dulbecco's modified Eagle's medium: nutrient mixture F-12 (DMEM/F-12). After becoming completely confluent, FITC-dextran (1 mg/mL) was then added to the upper chambers and samples from the lower and upper chambers were obtained at different time points (1, 3, and 6 h), and fluorescence intensity measurements were performed using a plate reader. The FITC-dextran that passed across the ARPE-19 cell monolayer was normalized, and *P*_*o*_ was calculated. The equation for *P*_*o*_ is [(F_l_/Δ*t*)V_A_]/(F_A_ *A*) whereas *P*_*o*_ is in cm/s; F_l_ is basolateral fluorescence, F_A_ is apical fluorescence, Δ*t* is the change in time, *A* is the surface area of the Transwell, and V_A_ is the volume of the basolateral chamber [29]. Similar experiments were performed with ARPE-19 cells treated with HG or NG for 5 days. In addition, one group was transfected with caspase-14 siRNA or the scramble siRNA before HG treatment. The knockdown of the caspase-14 was confirmed by Western blotting prior to initiating the experiment.

### 2.6. Measurement of the Transcellular Electrical Resistance (TER) by Electric Cell Substrate Impedance Sensing (ECIS)

Since RPE barrier dysfunction includes changes in the TER across the confluent monolayer, we evaluated the effect of modulating caspase-14 expression on RPE TER under NG or HG condition. ARPE-19 cells were transfected with caspase-14 siRNA or scrambled siRNA and placed into DMEM/F-12 medium with 1% FBS for 24 hours in 25 cm^2^ flasks. Once the cells were 90–95% confluent, ARPE-19 cells were seeded onto 8-well 10w3 + arrays (20,000 cells per well) and placed onto the ECIS machine (Applied Biophysics, Grand Island, New York). After 4 hours, the medium was changed to the NG or HG treatment in DMEM/F-12 medium and the measurements of TER were taken over 24 hours. Additional experiments were performed on ARPE-19 cells expressing caspase-14 following transfection with the caspase-14 or control plasmid.

### 2.7. Assessment of ARPE-19 Cell Cytoskeleton

ARPE-19 cells, which were previously transfected with the caspase-14 vector or the empty vector, were seeded in 8-well chamber slides (Lab Tek II) (about 2000 cells/well) for 24–48 h. Cells were then fixed with 4% paraformaldehyde, permeabilized for 5 min with 0.1% Triton X-100, and blocked with a 5% bovine serum albumin (BSA) (ACROS Chemical, Wyman, Massachusetts) for 30 min at room temperature. Diluted antivinculin 1 : 100 (Millipore, Billerica, Massachusetts) containing 5% BSA was added to the cells and incubated for 1 h at room temperature. Diluted goat anti-mouse FITC conjugated secondary (1 : 200) and TRITC conjugated phalloidin (1 : 200) (Millipore, Billerica, Massachusetts) were added to each well and incubated for 1 h at room temperature. Cells were incubated with DAPI solution (1 : 1000) (Millipore, Billerica, Massachusetts) for 5 minutes at room temperature. The slides were mounted using mounting media (Vector Laboratories, Burlingame, California) and images were obtained using immunofluorescence microscopy (LSM 510; Carl Zeiss, Thornwood, NY).

### 2.8. Measurement of the Activity of Caspase-1, -3, -4, -5, -8, and -9

ARPE-19 cells were transfected as previously described (with caspase-14 siRNA, caspase-14 plasmid, and control plasmid) and once the cells were 70% confluent the medium was changed to serum-free DMEM/F-12 medium for 24 h. The medium was then changed to HG or NG medium and the cells were grown in this medium for 5 days, and afterwards the cells were lysed. The cell lysates were collected and used to measure caspase activity by caspase family activity colorimetric II kit (Abcam, Cambridge, Massachusetts). This included the activity of caspase-1, -3, -4, -5, -8, and -9. The kit protocol was followed and the activity was measured by microplate reader at 400 nm.

### 2.9. Western Blotting

We evaluated the expression of caspase-14 and cleaved caspase-4 in RPE cells lysate. Confluent monolayers of ARPE-19 cultures were lysed using lysis buffer (5 mL of 1X RIPPA; Millipore, Billerica, Massachusetts) supplemented with proteinase inhibitor cocktail (Sigma-Aldrich, St. Louis, MO) and then scraped with a sterile scraper (Fisher Scientific, Wyman, Massachusetts). Cell lysate was incubated for 30 minutes on ice and then centrifuged at 14,000 rpm for 30 min at 4°C. The supernatant was collected for protein assay and the pellet was discarded. ARPE-19 cell lysates (50 *μ*g protein) were separated by sodium dodecyl sulfate-PAGE using a 10% ready precast gel (Bio-Rad, Hercules, CA), transferred to polyvinylidene fluoride membrane, and reacted with rabbit polyclonal caspase-14 antibody (Sc-5628; Santa Cruz Biotechnology, Santa Cruz, CA) followed by incubation with horseradish peroxidase-conjugated secondary antibody and examination by enhanced chemiluminescence (Amersham Pharmacia, San Francisco, CA). The membranes were then stripped and reprobed with *β*-actin to demonstrate equal loading, and the results were analyzed using Image-J program.

### 2.10. Assessment of Phagocytic Activity of RPE Cells

ARPE-19 cells were transfected with Lipofectamine 2000 according to the previously described protocol [27] with caspase-14 plasmid and control plasmid. Triplicates were seeded in 96-well plates at 100,000 cells/well in 1X DMEM medium supplemented with 1% FBS and 1% PSN. The medium (100 *μ*L) was placed into three wells for a no cell background. Once the cells adhered to the plate, culture medium was removed and replaced with pHrodo red phagocytosis particle (Invitrogen, Grand island, NY) (1 mg/mL) suspension. Immediately, the plate was transferred to 37°C incubator for 2 h. Fluorescence intensity was measured using a plate reader at an excitation wavelength 560 nm and an emission wavelength 585 nm.

### 2.11. TUNEL Assay

ARPE-19 cells that were previously transfected were grown in four-well chamber cells (Lab Tek 11) for five days in treatment medium. TUNEL assay (Promega, Madison, WI) was done according to the kit protocol. The nuclear stain propidium iodide was used to stain the nuclei of the cells on the chamber slides. The samples were analyzed under a fluorescence microscope and ten pictures per group were taken. The number of apoptotic cells relative to the number of normal cells in each microscopic field were counted using image J.

### 2.12. Statistical Analysis

Statistical analysis was done in GraphPad Prism 5. *t*-test was used to detect any significant difference between two groups and one-way ANOVA was used to detect any significant difference between 3 or more groups followed by Tukey analysis. Results are shown as mean ± SEM and were considered significant when *P* value < 0.05. At least 4 dishes were prepared for each treatment group and each experiment was replicated with at least 3 different batches of retinal cells.

## 3. Results

### 3.1. Effect of High Glucose on Caspase-14 Expression

**Figure 1:**
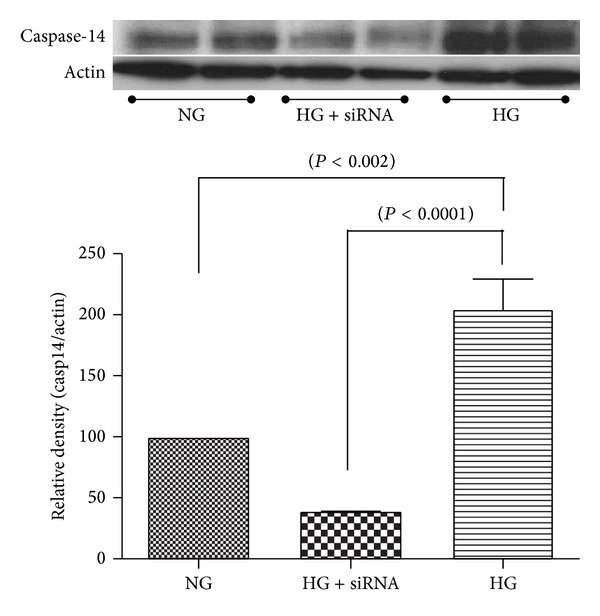
High glucose conditions increased caspase-14 expression in ARPE-19 cells. Western blot analysis of caspase-14 showed a significant increase in caspase-14 expression of RPE cells under high glucose (30 mM D-glucose) compared with normal glucose (5 mM D-glucose + 25 mM L-glucose) conditions. Transfection of ARPE-19 cells with caspase-14 siRNA significantly reduced caspase-14 in RPE cells under high glucose conditions (*n* = 4, *P < 0.05).

**Figure 2:**
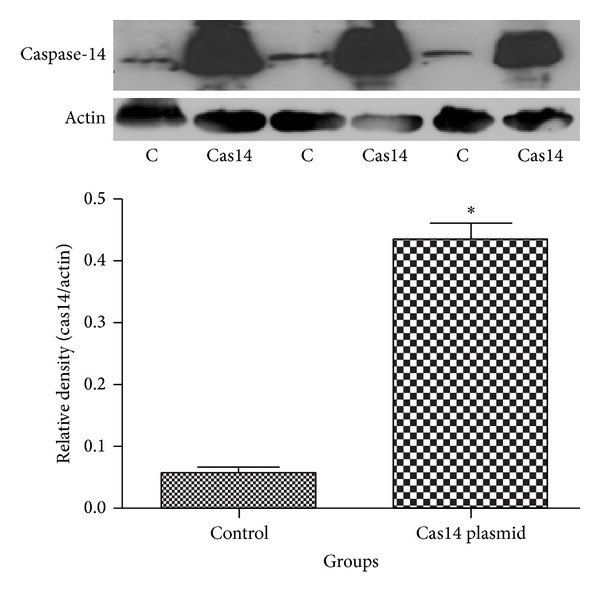
Overexpression of caspase-14 in ARPE-19 cells. Western blot analysis of caspase-14 in ARPE-19 cells transfected with pCMV plasmid encoding human caspase-14 cDNA showed a remarkable increase in the levels of caspase-14 compared with cells expressing the empty vector (*n* = 4; *P < 0.0001).

To test whether caspase-14 is implicated in hyperglycemia-induced RPE dysfunction, we first evaluated the changes in caspase-14 expression in ARPE-19 cells grown in HG (30 mM D-glucose) or in NG (5 mM D-glucose + 25 mM L-glucose) for 5 days. Western blot analysis of the cell lysates demonstrated that caspase-14 was significantly upregulated in ARPE-19 cells grown under HG conditions compared with NG. Transfection of the ARPE-19 cells with caspase-14 siRNA significantly abrogated caspase-14 expression under HG conditions (Figure 1). Caspase-14 expression was also evaluated in ARPE-19 cells transfected with caspase-14 plasmid or empty vector to assure the efficacy of the caspase-14 plasmid expression. Caspase-14 transfected ARPE-19 cells had significantly higher caspase-14 than cells transfected with empty vector (*P* < 0.0001; Figure 2).

### 3.2. Effect of Caspase-14 Expression on RPE Barrier Function

**Figure 3:**
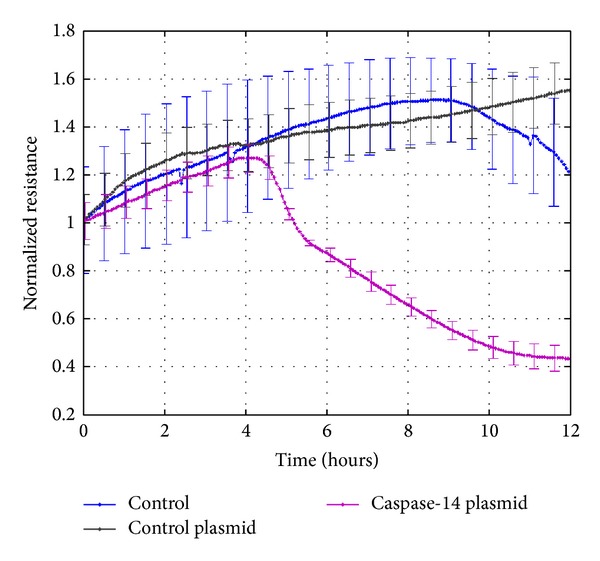
Effect of HG and caspase-14 expression on RPE barrier function. ECIS analysis of the transcellular electrical resistance (TER) demonstrated a significant decrease in the TER by caspase-14 expression compared to RPE cells transfected with or without the empty vector (*n* = 4, *P* < 0.05).

Breakdown of the RPE barrier function contributes to the development of DME, a major cause of vision loss in diabetic patients. Thus, we studied the effect of caspase-14 expression on the RPE cell barrier function by measuring the changes in the transcellular electrical resistance (TER) and stress fibers levels and organization. The zero time point is when the ARPE-14 cells reach full confluences and resistance was measured until the cells start detaching from the array. ARPE-19 cells transfected with caspase-14 plasmid had lower resistance compared with cells transfected with empty vector (Figure 3).

**Figure 4:**
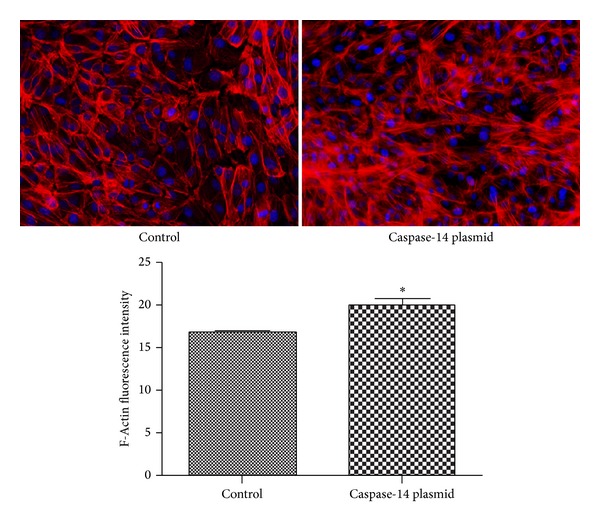
Immunofluorescence staining of RPE cell's cytoskeleton, F-actin (red). The nuclei were counterstained with DAPI (blue). Please note the marked increase and disorganization of the stress fibers (F-actin) immunoreactivity in caspase-14 expressing RPE cells compared with control cells (*P < 0.0008 versus control).

To further evaluate the impact of caspase-14 expression on RPE barrier function we examined the changes in the distribution and expression of ARPE-19 cell cytoskeleton protein F-actin, which is known to increase and become disorganized by hyperglycemia [30]. The relative distribution of ARPE-19 cell F-actin was monitored using phalloidin (red) staining, which binds to F-actin and provides details of the cellular cytoskeleton. There was a significant increase in the levels and the disorganization of F-actin stress fibers in caspase-14 expressing cells compared to control (Figure 4).

### 3.3. Effects of Caspase-14 Expression on RPE Cell Phagocytic Function

**Figure 5:**
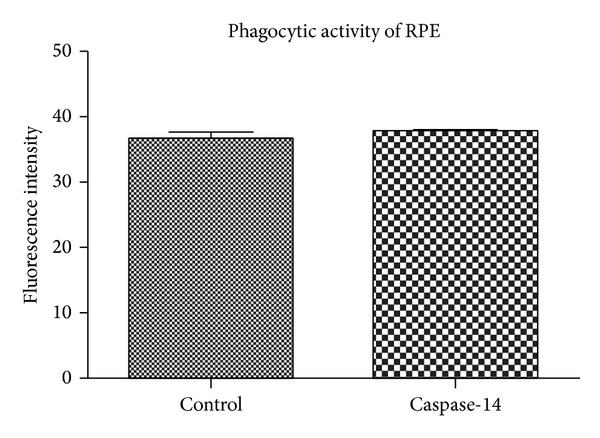
Effect of caspase-14 expression on ARPE-19 cell phagocytic activity. Assessment of phagocytic activity of the RPE cells was performed using a commercially available phagocytic assay kit. We observed no significant differences between the phagocytic activity of RPE cells expressing caspase-14 and the control (*P* > 0.05).

In addition to the role of RPE in maintaining the outer retinal barrier, RPE cells also play an important role in phagocytosis of the photoreceptor outer segments. Therefore, we tested whether caspase-14 effect is specific to the barrier function by examining the effects of caspase-14 expression on the phagocytic function of the ARPE-19 cells using a commercially available phagocytic assay kit. Our experiments demonstrated no significant differences in the phagocytic activity of caspase-14 overexpressing RPE cells compared with control cells (38 + 0.7 versus 37 + 1; Figure 5).

### 3.4. Effect of Caspase-14 Knockdown on HG-Induced RPE Hyperpermeability

**Figure 6:**
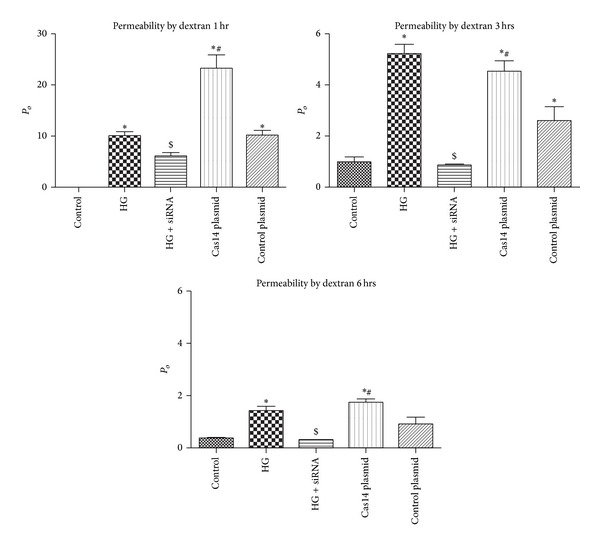
FITC-dextran flux assay. Permeability is defined by *P*_*o*_ (cm/s). There was a significant increase in permeability of cells cultured under high glucose (HG) conditions or transfected with caspase-14 plasmid at different time points (1, 3, and 6 h) compared with cells grown under normal glucose (NG) conditions or HG-treated cells transfected with caspase-14 siRNA (*P < 0.0001). Caspase-14 siRNA is significantly lower than high glucose at 3 h and 6 h (^$^ *P* < 0.001). Please note that caspase-14 plasmid has the same permeability effect as HG at 3 and 6 h (*n* = 4, *P < 0.05 versus control and HG + caspase-14 siRNA, ^#^ *P* < 0.05 versus control plasmid).

To assess the direct effect of caspase-14 transfection on RPE permeability compared to the control, we first examined whether transfection with the caspase-14 vector induces changes in FITC dextran flux through a confluent monolayer of ARPE-19 cell. We noticed that ARPE-19 cells became significantly permeable to FITC-dextran when transfected with caspase-14 compared with vector control cells after 1, 3, and 6 hr. HG treatment induced a similar effect as caspase-14 overexpression on the ARPE-19 cell permeability. However, this effect was significantly abrogated in ARPE-19 cells transfected with the caspase-14 siRNA compared with the scrambled control siRNA (Figure 6).

### 3.5. Caspase-14 Expression Is Implicated in HG-Induced Apoptosis of RPE Cells

**Figure 7:**
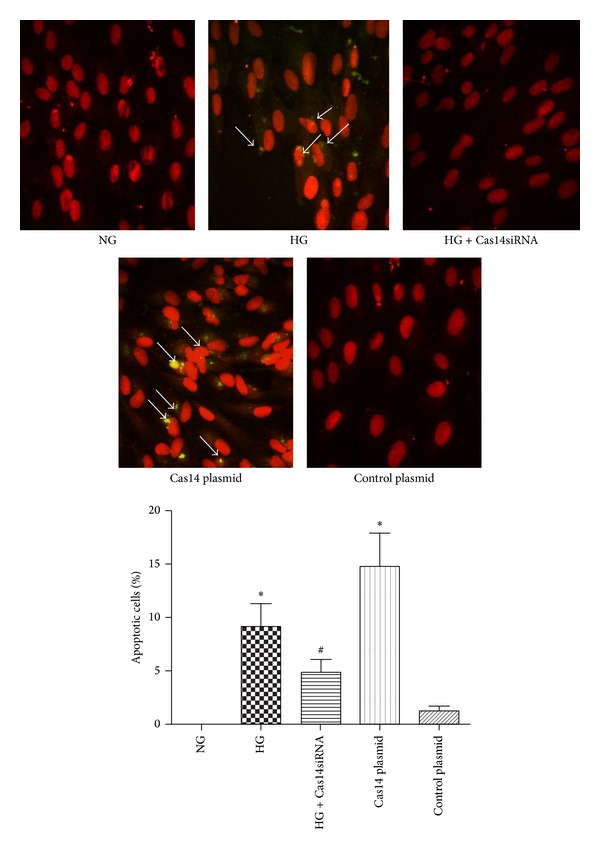
Increased apoptosis in RPE cells cultured under HG conditions or overexpressing caspase-14. Both high glucose treatment and caspase-14 transfected cells showed significantly increased levels of apoptosis compared with cells under NG or expressing control vector (*P < 0.001). Caspase-14 knockdown by siRNA reduced the number of apoptotic RPE cells under HG conditions. However, the number of apoptotic cells was higher than the control (^#^ *P* < 0.05 versus HG). Cell transfected with control plasmid had no significant difference in apoptosis compared with control (*n* = 4; *P* > 0.05).

The exact function of caspase-14 in various tissues other than skin has not yet been characterized. It is not clear if caspase-14 belongs to the proapoptotic or proinflammatory caspases. Therefore, it is important to examine if modulation of caspase-14 expression impacts these pathways in the RPE cells. For this purpose we measure the rate of apoptosis in ARPE-19 cells by TUNEL assay. High glucose conditions or caspase-14 overexpression significantly enhanced the apoptotic cell death of ARPE-19 cells compared to cells under NG or transfected with control plasmid, respectively (*P* < 0.001). Furthermore, caspase-14 knockdown reduced the number of RPE cells undergoing apoptosis under HG conditions (*P* < 0.05; Figure 7).

### 3.6. Effects of Caspase-14 Expression on the Activity of Other Caspases

**Figure 8:**
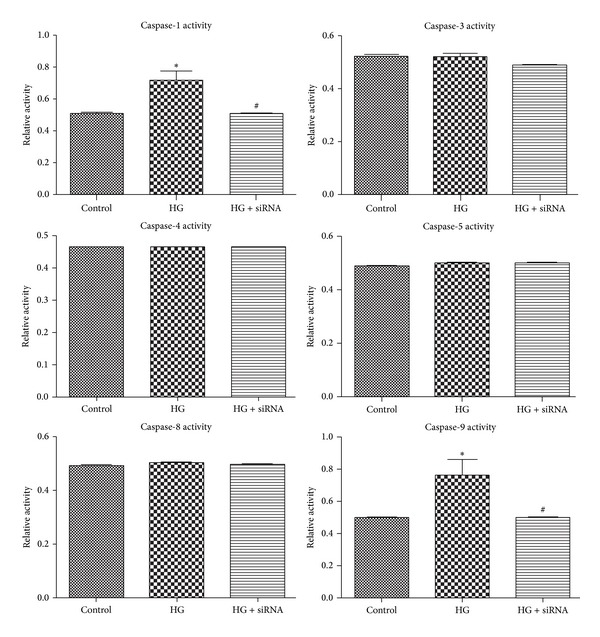
The effects of high glucose conditions on caspases activity. High glucose conditions increased the activity of caspase-1 and caspase-9 compared with NG. The siRNA knockdown of caspase-14 prevented the effect of HG conditions on activity of these caspases (*P < 0.05 versus control and caspase-14 siRNA, ^#^ *P* < 0.05 versus high glucose). There were no significant changes in the levels of caspase-3, -4, -5, or -8 activity under the experimental conditions utilized here (*n* > 3; *P > 0.05).

Since there is no information regarding how caspase-14 interacts with other members of caspase family, we sought to test the impact of caspase-14 knockdown on HG-induced activation of various caspases in ARPE-19 cells. HG increased the activity of caspase-1 and caspase-9 out of several caspases examined including caspase-3, -4, -5, and -8. Caspase-14 knockdown significantly reduced the activation of caspase-1 and caspase-9 under HG conditions (Figure 8).

## 4. Discussion

To the best of our knowledge, the current study is the first to investigate the impact of caspase-14 expression on RPE cell barrier and phagocytic function. Our major findings are as follows: (1) hyperglycemia upregulates caspase-14 expression in human RPE cells, (2) caspase-14 expression impairs RPE barrier function with no effect on its phagocytic function, and (3) caspase-14 knockdown in RPE inhibits hyperglycemia-mediated RPE barrier disruption, activation of caspase-1 and -9, and enhanced apoptosis.

Our previous study demonstrated that caspase-14 is normally expressed in the retina and various retinal cells including RPE cells and was upregulated in human and mouse retina during diabetes. Additionally, the overexpression of caspase-14 demonstrated a proapoptotic effect in cultured retinal endothelial cells and pericytes suggesting caspase-14 as a potential player in the pathogenesis of DR via enhancing retinal vascular cell death and capillary degeneration, which causes loss of inner retinal barrier function [27, 31]. Here we investigated whether caspase-14 also contributes to hyperglycemia-induced RPE barrier dysfunction, which constitutes the outer retinal barrier.

The RPE is the major component of the outer BRB, playing important roles in the flow of metabolites and ions from the choroidal blood supply to the neural retina [22, 32], and contributes to the ocular vascular homeostasis through production of pro- and antiangiogenic factors. Therefore, RPE may provide good target for studying molecular basis of DR and diabetic macular edema (DME); in particular, hyperglycemia has been shown to induce inflammation and apoptosis and disrupts RPE tight junctions leading to breakdown of RPE barrier and finally DME [33, 34].

Caspase-14 was found to be expressed in tissues involved in barrier function such as epidermis and RPE and to preserve skin barrier and protect it against dehydration and ultraviolet light [34, 35]. However, the current study found that, similar to hyperglycemia, overexpression of caspase-14 leads to hyperpermeability of RPE cells. Transmembrane proteins and cytoplasmic protein of RPE cells are linked to the actin cytoskeleton and participate in many important cellular processes, such as cell motility, phagocytosis, and establishment and maintenance of cell junctions and cell shape [10]. In this study, we found that overexpression of caspase-14 transfection was accompanied by disorganization of RPE cytoskeleton including increased amounts of F-actin filaments.

These data suggest that caspase-14 is implicated in RPE barrier function and could be a potential molecular target to study the underlying mechanisms of retinal diseases associated with disruption of the outer retinal barrier such as DME. This led us to test the effects of caspase-14 knockdown in RPE cell's hyperglycemia-induced barrier dysfunction. Consistent with our hypothesis, knockdown of caspase-14 preserved RPE cell barrier function under HG conditions.

**Figure 9:**
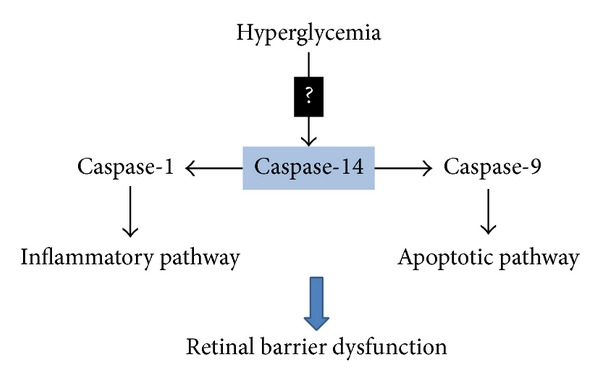
Schematic diagram demonstrates the proposed role of caspase-14 in the hyperglycemia-induced RPE barrier dysfunction and its potential role in DME. Hyperglycemia upregulates caspase-14 level/activity in the RPE cells modulating the activity of both caspase-1 and caspase-9 and promoting the proinflammatory and proapoptotic responses to hyperglycemia, respectively.

Caspase-14 is mainly involved in the epithelial differentiation, which shares some features with apoptosis including DNA fragmentation, nuclear condensation, and activation of caspase-3 [36]. The proteolytic process of procaspase-14 has been reported to increase in brain following reperfusion injury, and this was linked to increased number of neuronal cell deaths [25]. Our previous data showed that activation of caspase-14 under pathological conditions might influence retinal vascular function by promoting apoptosis of retinal microvascular cells [27]. RPE cells are believed to actively participate in progression of many inflammatory diseases including DR through caspase-mediated inflammatory and apoptotic pathway mechanisms [20, 31]. These include activation of various caspases including caspase-1, caspase-4, and caspase-3. Therefore, we determined how caspase-14 interacts with other caspases. For this purpose we examined the effect of caspase-14 knockdown on HG-mediated activation of other caspases in RPE cells, especially the known caspases which impact RPE cell function including caspase-1, -3, -4, -5, -8, and -9 [37, 38]. HG conditions significantly increased activities of caspase-1 and caspase-9 with no effect on other tested caspases. The effect of HG conditions on caspase-1 and caspase-9 activities was attenuated by caspase-14 knockdown. These results suggest that caspase-1 and caspase-9 are the primary caspases implicated in hyperglycemia-induced RPE dysfunction, and caspase-14 is upstream of caspase-1 and caspase-9 in these processes. Pyroptosis is a proinflammatory mode of cell death, whereas apoptosis is noninflammatory programmed cell death which occurs in RPE cells and mediated by the caspase-1 rather than apoptotic caspases such as caspase-3 [39–41]. On the other hand, caspase-9 is involved in the activation cascade of other caspases [42, 43] and apoptosis [44, 45]. Thus, our data implicate caspase-14 in hyperglycemia mediated RPE barrier dysfunction and apoptosis. This occurs in a caspase-3 independent manner and may involve activation of caspase-1 and caspase-9 dependent inflammatory and proapoptotic pathways, respectively (Figure 9).

To evaluate whether the function of caspase-14 is limited to the barrier function of the RPE cells, we also tested the impact of caspase-14 on their phagocytic activity. Interestingly, we noticed no changes in the phagocytic activity of RPE cells by caspase-14 expression. Collectively, our findings indicate caspase-14 as a potential player in retinal diseases associated with RPE barrier dysfunction such as DME. This process may constitute a novel mechanism for the pathogenesis of DME and, in turn, a novel therapeutic target to treat these pathological conditions. Further studies are required to identify the mechanisms of caspase-14 regulation and its coordinated interactions with other caspases and apoptotic proteins in retina.
